# Critical View on the Qualification of Electronic Tongues Regarding Their Performance in the Development of Peroral Drug Formulations with Bitter Ingredients

**DOI:** 10.3390/pharmaceutics16050658

**Published:** 2024-05-15

**Authors:** Denise Steiner, Alexander Meyer, Laura Isabell Immohr, Miriam Pein-Hackelbusch

**Affiliations:** 1 Institute of Pharmaceutical Technology and Biopharmaceutics, University of Muenster, Corrensstraße 48, 48149 Muenster, Germany; denise.steiner@uni-muenster.de; 2 Institute for Life Science Technologies (ILT.NRW), Ostwestfalen-Lippe University of Applied Sciences and Arts, Campusallee 12, 32657 Lemgo, Germany; 3 NextPharma Germany Bidco GmbH, Hildebrandstrasse 12, 37081 Goettingen, Germany; isabell.immohr@nextpharma.com

**Keywords:** e-tongues, performance, qualification, taste masking, bitterness, dosage forms, drug formulations

## Abstract

In this review, we aim to highlight the advantages, challenges, and limitations of electronic tongues (e-tongues) in pharmaceutical drug development. The authors, therefore, critically evaluated the performance of e-tongues regarding their qualification to assess peroral formulations containing bitter active pharmaceutical ingredients. A literature search using the keywords ‘electronic’, ‘tongue’, ‘bitter’, and ‘drug’ in a Web of Science search was therefore initially conducted. Reviewing the publications of the past decade, and further literature where necessary, allowed the authors to discuss whether and how e-tongues perform as expected and whether they have the potential to become a standard tool in drug development. Specifically highlighted are the expectations an e-tongue should meet. Further, a brief insight into the technologies of the utilized e-tongues is given. Reliable protocols were found that enable (i) the qualified performance of e-tongue instruments from an analytical perspective, (ii) proper taste-masking assessments, and (iii) under certain circumstances, the evaluation of bitterness.

## 1. Introduction

In recent years, the development of drug formulations that meet the needs and preferences of patients has increasingly been focused on. This was emphasized by regulatory authorities, for example, in the guideline on pharmaceutical development of medicines for pediatric use. Therein, a verification of the acceptability of a new pediatric formulation prior to its approval is demanded [1]. The Pediatric Regulation of 2006 also calls for the development of suitable formulations for children, but without subjecting them to unnecessary clinical trials [2].

Acceptable and suitable formulations ensure good compliance of patients. Therefore, drugs are often formulated as peroral (solid) dosage forms, as these are typically preferred by patients [3]. However, a possible bitterness of the (active) ingredient and, therewith, an unpleasant taste, has to be clarified in advance to develop an acceptable and suitable taste-masked peroral dosage form. Information about the bitterness of substances is difficult to obtain, as, during the pharmaceutical drug development process, the most relevant criteria are, indisputably, safety, efficacy, and quality. For safety reasons, taste studies in patients, especially of new chemical entities (NCEs), are limited. However, regulators are aware of and accept results from these tests [4], which can be part of a Phase 1 dose-escalation study [5]. Since the availability of such data is limited, a need for qualified non-human taste-assessment tools remains.

This is why, in recent years, various methods have been developed, including animal gustatory tests, cell-based assays, and electronic taste sensing. Animal gustatory tests have predominantly been conducted with rats [6]. Further, fish and drosophila models have also been studied, as these species provide similar taste receptors as mammals [7,8]. In addition to ethical considerations, animal-based gustatory methods are time-consuming and only accessible to a limited extent. Further, the results obtained can only indirectly prove human taste preferences [6,9]. Cell-based assays, such as calcium imaging, are in vitro methods that allow for a risk-free evaluation of substances with high toxicity. Again, these results also only serve as indirect indicators of the taste of a substance [9].

Instruments providing quantitative relations between sensor signals and drug concentrations in a reproducible way without any safety concerns are analytical taste-sensing systems. While different terms are used for these sensing systems, such as taste chip, taste sensor, electronic sensor array system, or biomimetic sensor array system [10], in the pharmaceutical context they are usually referred to as ‘electronic tongues’ or ‘e-tongues’. Over the last two decades, the applicability of these e-tongues as a versatile tool in the development of taste-masked drug formulations has made significant progress. Following good manufacturing practices (GMPs), however, any instrument applied in pharmaceutical drug development must be qualified. A proper qualification process comprises design qualification (DQ), installation qualification (IQ), operational qualification (OQ), and performance qualification (PQ) [11]. The DQ is, thereby, the documented verification that the instrumental design is suitable for its intended task. In the case of e-tongues, users expect these instruments to provide information about the (bitter) taste of a substance and/or about the taste-masking success of a formulation containing a bitter drug. To fulfill these requirements, a sound sensor design, which is discussed in the ‘Theoretical Background’ (Section 3.1) of this review, is required. With instruments that have successfully been qualified regarding their design (passed the DQ), an IQ is to be carried out prior to operation. This ensures that the e-tongues are installed correctly and function in accordance with their specifications [11]. Although this part of the qualification process is also essential, it is not covered further in this review, as it has, to the best of the author’s knowledge, not been critically discussed in the literature. The equally mandatory OQ comprises functional testing of the instrument, including operator advice, and can be a feature of scheduled quality assurance testing. In this context, the precise performance of the sensing unit of e-tongues must be proven prior to any analytical task. In this review, this topic is briefly discussed in the ‘Theoretical Background’ (Section 3.2).

Although each of the four mentioned qualification steps is essential, this review strongly focuses on the discussion of the PQ aspects. The authors aim to critically discuss, whether e-tongue instruments ‘perform as expected under real conditions’ and, therefore, whether they have the potential to become a standard tool in drug development. To answer this question, publications of the past decade were reviewed, specifically highlighting the expectations an e-tongue should meet. Authors therefore differentiated the ‘performance qualification’ to (a) the performance of e-tongue instruments from an analytical perspective, (b) their usability for proper taste-masking assessments, and (c) their ability to evaluate the bitterness of a (new) drug substance.

## 2. Research Strategy

This review aims to highlight the advantages, challenges, and limitations of electronic tongues (e-tongues) in pharmaceutical drug development. Specifically, the authors critically evaluated the literature with a view to the qualification of the e-tongue’s ability to assess peroral formulations containing bitter ingredients properly. The authors therefore differentiated the PQ into the following categories: (a) the performance of e-tongue instruments from an analytical perspective, (b) their usability for proper taste-masking assessments, and (c) their ability to evaluate the bitterness of a (new) drug substance.

A literature search using the keywords ‘electronic’, ‘tongue’, ‘bitter’, and ‘drug’ in a Web of Science search was initially conducted. The growing interest in this technology becomes obvious, as between 2004 and 2013 only 16 publications were listed, while, since 2014, the Web of Science lists 64 publications, thereof, 55 research papers (excluding reviews) deal with e-tongues. E-tongue technology primarily relies on electrochemical methods, including potentiometry [12,13], voltammetry [14,15], or impedance (spectroscopy) [16,17], but it can also include mass-sensitive (piezoelectric sensors) [18] or optical devices [19]. Based on the underlying literature analysis, over the past decade, e-tongue systems utilized for pharmaceutical analysis have predominantly employed electrochemical sensors (Table S1). In summary, 55 publications utilized 57 e-tongues. Among these, the Insent taste sensing systems (TS5000-Z, SA402B Insent Inc., Atsugi-Shi, Japan) and the ASTREE liquid and taste analyzer (AlphaMOS, Toulouse, France) were used 26/57 (Insent Inc. taste sensing systems) or 17/57 (AlphaMOS ASTREE liquid and taste analyzer) times, respectively, accounting for a combined 75%. Laboratory prototype nonspecific, low-selective potentiometric e-tongues were used in 9/57 (16%), electrical impedance spectroscopy (EIS)-based e-tongues in 2/57 (4%), and a voltammetry-based e-tongue, a laboratory prototype taste biosensor (rat cardiomyocytes + microelectrode assay), and the ctongue (Shanghai Baosheng, Shanghai, China) were each used once (5% combined). The authors are aware that using different keywords and reference data bases may yield different percentages. However, the application of commercially available systems from Insent and AlphaMOS, along with the laboratory prototype non-specific, low-selective potentiometric e-tongues, will most likely remain prominent for the addressed task. Hence, the focus of this review primarily revolves around these potentiometric e-tongue systems.

Taking the publications of the past decade (2014–2023, Table S1) as a basis, the authors discuss whether and how e-tongues perform as expected and whether they have the potential to become a standard tool in drug development. Following the idea of a critical review, further information and insights into additional publications were provided, wherever necessary.

## 3. Theoretical Background

### 3.1. About the Design Qualification of E-Tongues

According to a technical report of the International Union of Pure and Applied Chemistry (IUPAC), e-tongue instruments consist of nonspecific, low-selective (chemical) sensor arrays that act to produce ‘analytically useful signals’ for the analysis of multicomponent matrices [20]. The interaction between the sensor arrays and sample solutions generates a sample-specific sensor ‘spectrum-like’ response pattern. Through the application of appropriate mathematical evaluation methods, such as pattern recognition or multivariate data analysis, these high-dimension signals produced by the sensor arrays can be processed properly [20]. As mentioned in the introduction, the DQ is the documented verification that the instrumental design is suitable for its intended task. In the following, the theoretical background of different e-tongue technologies will be presented, whereby the focus lies on the design for the intended task, to provide information about the taste of a substance and/or about the taste-masking success of a formulation.

#### 3.1.1. Potentiometry-Based Electronic Tongues

Potentiometry is the underlying measurement principle of many e-tongue types. This method is based on measuring the electromotive force in the galvanic cell. Such an electrochemical cell consists of a reference electrode with constant potential, typically a reference electrode as the Ag/AgCl electrode, and a working electrode, also referred to as a potentiometric sensor. The electromotive force is thus the difference between two electrode potentials. In the case of potentiometric e-tongues, a set of different potentiometric sensors is often applied. This set-up of one reference electrode and several potentiometric sensors is also called a sensor array [21,22]. The potentiometric sensors generate a concentration-dependent potential through the ad- or absorption of dissociated target analytes. This process involves the formation of an ion concentration gradient across a semi-permeable membrane, generating a potential.

The concentration or activity of the analyte can be determined by the Nernst and Nikolsky equation, which describes the relationship between the sensor responses and the activity of the analyte [23,24]. Unlike ion-selective electrodes (ISEs), such as the glass electrode, low selectivity is desired for e-tongue sensors (Table 1). This allows chemically similar groups, such as alkaloids, to be detected in a similar manner by the same membrane electrode. If certain taste sensations that can be caused by different groups of molecules are assigned in this way [25,26,27], low selectivity is also referred to as global selectivity in the literature. Low selectivity is generally used to describe cross-selectivity, where each sensor responds to a variety of tastants [13,20]. This property is essential for the use of such low-selective electrodes for taste(-masking) studies. The aim of these studies is not to selectively quantify individual substances, but rather to identify possible taste impressions from similar analytes.

In 1990, the first e-tongue based on a so-called taste sensor with global selectivity was introduced [28]. Since then, the potentiometric multichannel taste sensor systems, which are composed of lipid membrane sensors, have been further improved and also commercialized. Available instruments include the Insent taste-sensing systems SA401, SA402, SA402B, and TS5000-Z (Insent Inc., Atsugi-Shi, Japan). Initially, the commercially available sensors of these systems have been developed based on grouped signals for key substances in human taste perception [29]. These PVC membrane sensors, which deliberately do not incorporate selective ion carriers (ionophores), in order to generate a more generic response, consist of various types of artificial lipids, such as tetradodecylammonium bromide, trioctylmethylammonium chloride, or oleic acid. Further, plasticizers, such as dioctyl phenyl-phosphonate, 2-nitrophenyl octyl ether, or bis(1-butylpentyl) adipate are incorporated. These components are mixed with polyvinylchloride (PVC) as an immobilizing polymer [29].

This results in sensors dedicated to the bitterness of cationic bitter substances (e.g., protonated alkaloids; commercial sensors are labeled as SB2AN0, SB2AC0, SB2BT0), the bitterness of anionic bitter substances ((e.g., deprotonated isoalpha acid, commercial sensor is labeled as SB2C00), as well as to the sourness (SB2CA0), sweetness (SB2GL1), and saltiness (SB2CT0) of dissolved analytes. Further, the taste impressions of umami (SB2AAE) and astringency (SB2AE1) can be assigned by the sensors [30]. As described above, the low selectivity of the membrane electrodes is desirable so that the sensors can react to different components of a sample solution.

The mechanism of the taste sensor response is explained by the Gouy–Chapman theory [31] and is based on the formation of an electrical double layer on a charged membrane. In terms of the sensor membrane composition, when the sensor is immersed in an aqueous solution, distinct membrane potentials are generated for different analytes due to the electrical double layer [29].

The first sensor arrays pointing towards nonspecific, low-selective sensors were introduced in the mid-1990s through a collaboration between a Russian and Italian working group [32]. The development of an e-tongue emerged from the idea to enhance the analytical application of highly selective sensors by less selective and cross-sensitive sensor arrays combined with an appropriate data analysis approach [33]. The chemically different potentiometric sensors can be prepared using diverse materials, such as chalcogenide glasses with added metals, or comparably to those of the Insent taste sensing systems mentioned above. Accordingly, membranes are composed of PVC as the polymer and combined with a plasticizer and ionophores [34], or other ionic additives for active recognition of sample molecules [35]. Ionophores can be ion exchangers or macrocyclic compounds, which can surround the target ions in a cavity-like environment [35]. As the selectivity of an ion-selective electrode (ISE) can be defined by the binding strength between the ionophore or other ionic additives to the target ion [35], the use of less selective ionophores can change an ISE to an electrode with cross-sensitivity towards a wide range of species with different sensitivity. Possible membrane materials, types of plasticizers, ionophores, and ionic additives are numerous, which allows for the preparation of individual membrane electrodes for various applications.

The potentiometric sensor of the commercially available e-tongue ASTREE liquid and taste analyzer (AlphaMOS, Toulouse, France) is based on chemically modified field-effect transistor technology (ChemFET). This technology is similar to the ISFET (ion-selective field effect transistor) technology, which was established in the 1970s [36] and is based on an ion-selective membrane attached to the gate region of a FET transistor. Since the compositions of the ion-selective membranes can be of a large variety, sensor arrays with low selectivity, also described as overlapping selectivity, can be produced. The basic principle of ISFETs combines the technologies of ISEs and solid-state microelectronics, whereas the metal gate of a conventional MOSFET (metal oxide semiconductor FET) is replaced by the ion-selective membrane [37]. The gate potential is therefore a result of the interaction between the membrane and the ions in solution. A potential is then applied to the drain, which causes a current to flow between the drain and the source depending on the charge of the semiconductor surface, which is controlled by the gate potential [38]. The employed working electrodes of the ASTREE liquid and taste analyzer (AlphaMOS, Toulouse, France) comprise a silicon FET coated with distinct organic materials [38]. Specialized sensor sets for food and pharmaceutical applications are available (the pharmaceutical sensor set with sensors labeled ZZ, AB, GA, BB, CA, DA, and JE, the food sensor set with sensors labeled SRS, GPS, STS, UMS, SPS, SWS, and BRS [39]), as well as one set for bitterness-intensity measurement [40]. The sensor sets differ in the composition of the silicon transistor coatings responsible for the electrochemical recognition of liquid samples. Thereby, sensors with good repeatability, sensitivity, and selectivity were developed [41]. Even though the compositions of the sensors are not fully reported, it is known that they also consist of a polymer matrix, a plasticizer, and several ionophore-like substances to ensure the electrochemical sensitivity of the sensors [42]. Each sensor set consists of seven cross-selective working electrodes with each sensor responding to the five basic tastes, sourness, sweetness, bitterness, saltiness, and umami, at different intensities [41].

**Table 1:** Explanation of the wording used by different authors to describe sensor characteristics. Symbols and colors are surrogates for different analytes, which are more or less different/comparable in terms of chemical behavior. The arrow and marking illustrate a possible detection.

Wording	Explanation	Scheme/Figure	Reference, e.g.,
Discrete selectivity	Sensors are selective towards one specific ion species	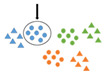	[32,35]
Low selectivity	Sensors are selective towards a number of different analytes	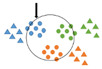	[25,29]
Overlapping selectivity			[38]
Cross-selectivity	Applied wording particularly for ASTREE liquid and taste analyzer (AlphaMOS, Toulouse, France), but also in the context of other e-tongues		[15,20,33]
Cross-sensitivity	Sensors show responses to a number of different analytes with distinguishable and reproducible sensor signals	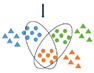	[15,33]
Global selectivity	Sensors respond consistently to the same taste species Basis of Insent taste sensing systems (Insent Inc., Atsugi-Shi, Japan)	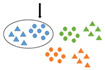	[25,29]

As most references for this review present and discuss results for the aforementioned e-tongue systems, voltammetry- and electrochemical impedance-based e-tongues will now shortly be presented. Further information regarding those methodologies can be found, amongst others, in [43].

#### 3.1.2. Voltammetry-Based Electronic Tongues

The e-tongue sensors described above are limited to ions as detectable compounds. The related potentiometric measurements are, as such, sensitive to noise and electromagnetic field interferences [14]. To overcome these drawbacks, in 1997 it was described ‘how various voltammetric techniques can be applied to generate information when combined with a multi-variate method’ [44]. Other than potentiometric methods, which are referred to as static (passive) methods in electroanalysis, voltammetry belongs to the group of dynamic (active) methods. In particular, voltammetry is a voltage-controlled technique. It includes all methods that are based on current–voltage measurements on stationary and solid working electrodes, regardless of their material composition. In contrast to potentiometric determinations, reduction or oxidation reactions are necessary to generate signals.

The applied potentiostats enable the potential to be controlled, for example, through a programmed voltage shift (V/s). The current measured from a certain potential in the scanned potential range, which is defined as the Faraday current, is generated and measured by a reduction or oxidation reaction on the electrode surface [45]. This reaction depolarizes the working electrode. The evaluation of such voltammetric determinations is made possible by the recorded current–voltage curves. Thereby, the potential at which the redox reaction begins and a current is measured represents the qualitative characteristic of the analyte(s), and the measured current intensity represents the quantity of the analyte(s). The potentials at which the current flow through the reduction or oxidation is measured are mainly influenced by the nature of the considered species. Further, the potentials are largely determined by the sample matrix. Thus, the potentials at which a change in current flow becomes measurable are only analyte-specific signals if largely identical matrices are used. This is why such determination methods are generally not transferable to other matrices and are much more selective than potentiometric e-tongues. How the applied potential is changed and how the current is measured distinguishes the different voltammetric methods [46].

Modern voltammetric electrochemical cells require three electrodes: a counter (auxiliary) electrode, a smaller working electrode that limits the current flow of the redox reaction through the very small electrode surface, and a reference electrode. This reference electrode is the reference electrode for setting the potential setpoint, no electricity flows through it [45]. The measured current flows between the working and counter electrodes. The resulting voltammograms exhibit indistinct bands containing a wealth of information. Thus, a single voltammetric working electrode can be equivalent to an e-tongue sensor array.

Conventionally, the working electrodes consisted of noble metals, e.g., Au, Pt, Ir, Pd, Rh, Ag, etc., but also of other metals, such as Re, Cu, Ni, Co, W, and Ti. Nowadays, carbon paste electrodes, graphite-epoxy composites, and screen-printed electrodes based on different materials and inks have been developed [46]. The latter approach allows for printing the working, reference, and counter (auxiliary) electrodes onto the same strip, even including many working electrodes on the same miniaturized device. Furthermore, molecularly imprinted polymer-based sensor arrays were presented showing high sensitivity and selectivity together with a wide detection range in the simultaneous determination of different drug substances [47]. Altogether, this allows for the development of individualized measurement systems.

Further, as stated above, voltammetry operates non-selectively in complex solutions, which is a requirement for its usability as an e-tongue technique. As further benefits, it is widely recognized for its high sensitivity, adaptability, simplicity, and robustness [14]. Currently, linear sweep voltammetry or cyclic voltammetry are the most common voltammetric techniques for e-tongues. Pulse voltammetric methods, e.g., differential pulse voltammetry (DPV) and square wave voltammetry (SWV), have also widely been used, and stripping techniques (anodic, cathodic, adsorptive) have been considered as e-tongue technologies [46].

#### 3.1.3. Electrochemical Impedance-Based Electronic Tongues

Electrochemical impedance spectroscopy (EIS) allows for determining the alternating current resistance (impedance) of electrochemical systems as a function of the frequency of an alternating voltage or alternating current. For all impedance methods, a sinusoidal excitation signal with a small amplitude is applied to the system under investigation. The response is measured, which can be a current, a voltage, or another signal of interest [48].

There are two different forms of representation for the impedance spectra obtained from EIS. The Nyquist diagram provides a quick overview and some qualitative interpretations [49]. The disadvantage of the Nyquist diagram is that the frequency information is not available. The more complete representation is a function of frequency in the form of two graphs, collectively known as a Bode plot (impedance curve and the phase shift).

EIS is currently a widely used technique for characterizing the behavior of complex electrochemical systems. The specialty of EIS is its ability to isolate and distinguish the influence of various physical and chemical phenomena at a given applied potential. This is often not possible with ‘traditional’ electrochemical techniques and explains why EIS is frequently used in the study of a number of complex systems. For several years, EIS has further been used to study semiconductor interfaces and the diffusion of ions through membranes [49].

The literature states that EIS is superior to other electrochemical techniques in its ability to provide a wealth of information for various electrical, electrochemical, and physical processes of real electrochemical systems [50]. This can be particularly challenging, as underlying processes might exhibit different timing behaviors. EIS now allows for the deconvoluting of a complex electrochemical system in individual processes with different time constants. The rationale behind this is that this technique works in the frequency domain over a wide range of frequencies (from 10 MHz to 0.1 Hz). So, in relation, low frequencies are used to analyze slow processes, while high frequencies are used to analyze fast processes [50]. By analyzing the impedance response, it is further possible to extract information about various properties of the solution, such as its composition, conductivity, and viscosity. However, a big inconvenience with impedimetric e-tongues is that they require a much slower, sequential approach for parallel measurements, which may be a drawback, for example, in transient events.

In 2003, this technique was combined with the unique properties of thin (Langmuir-Blodgett) films of conducting polymers with a lipid-like material and introduced as a taste-sensor technique [17]. To obtain resulting capacitances for taste-masking assessment, a single frequency [51], but also a spectrum of frequencies ranging from 1 Hz to 1 MHz at a constant potential, can be applied [16]. The applied frequencies comprise areas where the impedance of the equivalent circuit is dominated by the double-layer effect (<50 Hz), at which the properties of the coated electrodes are prominent (102 Hz–104 Hz) and the impedance is dominated by the geometric capacitance (>105 Hz) [17].

#### 3.1.4. Different, Different, but Same…?

Since the aforementioned e-tongue sensor systems differ in their designs, different performances related to taste-masking assessment are to be expected. An interlaboratory experiment aimed at comparing the performances of six different e-tongues to assess the same sample sets [52]. Therefore, the samples were analyzed in different laboratories with different e-tongues (TS-5000Z from Insent; αAstree from AlphaMOS; two potentiometric systems from Warsaw University of Technology, operating in flow and static modes; one potentiometric system from St. Petersburg State University/Laboratory of Artificial Sensory Systems, ITMO University; one voltammetric system from Universitat Autònoma de Barcelona), followed by a joint data evaluation by the group. As a result, it can be stated that the differences in performance were only minor and could be related to the different working principles. It can, furthermore, be stated that the differences between the results of e-tongues of different designs, as evaluated in [52], were not more pronounced compared to the differences published in a comparative study with four e-tongues from the same provider [53]. The latter differences were attributed to the different histories of the applied sensors. This highlights the importance of a sound PQ, which will be addressed in ‘Results and Discussion’ (Section 4).

### 3.2. About the Operational Qualification of E-Tongues

The mandatory OQ comprises functional testing of the instrument, including operator advice, and can be a feature of scheduled quality assurance testing. In this regard, the precise performance of the sensing unit of e-tongues must be proven prior to any analytical task. To achieve stable sensor responses requires a certain experimental design and measurement time. Thus, this review shortly discusses the operational set-up of the commercially available systems ASTREE liquid and taste analyzer (AlphaMOS, Toulouse, France) and Insent taste sensing systems (Insent Inc., Atsugi-Shi, Japan), which is necessary in order to evaluate and discuss the following performance of the applied e-tongues. Laboratory-based prototype nonspecific, low-selective potentiometric e-tongues could differ from this procedure [54].

**Figure 1:**
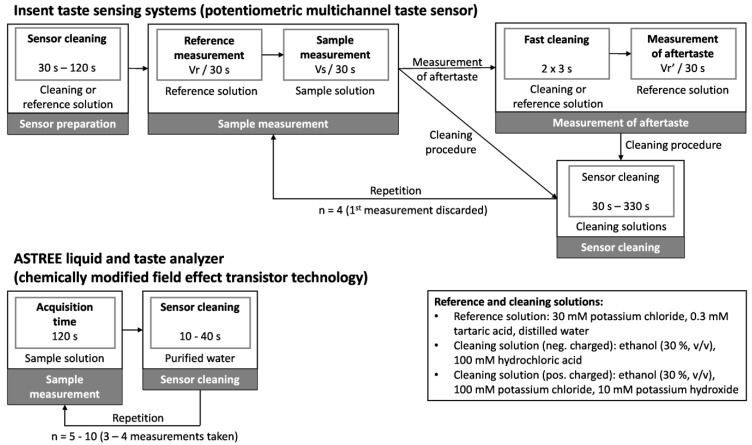
Measurement procedure of Insent taste sensing systems (Insent Inc., Atsugi-Shi, Japan)and ASTREE liquid and taste analyzer (AlphaMOS, Toulouse, France).

Focusing on the Insent taste sensing system (Insent Inc., Atsugi-Shi, Japan) first, prior to each actual measurement campaign, it is beneficial to condition the sensors in the standard solution for 24 h [55]. The actual measurement procedure starts with a cleaning step (Figure 1).

The sensors are washed either in the standard reference solution or in specific cleaning solutions. The latter are alcohol-based and have been selected particularly for the various lipid membrane compositions. An aqueous ethanolic solution (30% (*v*/*v*)) containing 100 mM hydrochloric acid is used for sensors with membranes containing negatively charged lipids, while an aqueous ethanolic solution (30% (*v*/*v*)) containing 100 mM potassium chloride and 10 mM potassium hydroxide is used for sensors containing positively charged lipids [30,55]. The measurement cycle starts with a reference measurement, whereby the sensors analyze a reference solution for 30 s. Afterwards, the sensors are dipped into the first sample solution for 30 s. Typically, one sample is measured four times. The supplier, however, recommends discarding the first measurement to allow for the conditioning of the sensors [30]. The change of membrane potential due to adsorption (referred to as CPA value or ‘aftertaste’) can also be measured. Therefore, the sensors are only briefly cleaned after the sample measurement itself before being immersed into a fresh beaker of the reference solution. The remaining potential (CPA) is then measured for an additional 30 s [30]. The sensor signals are calculated by the software by subtracting the measured sensor signals (Vs and Vr′) in volts from the signals measured in the reference solution (Vr).

The ASTREE liquid and taste analyzer (AlphaMOS, Toulouse, France) requires a sample acquisition time of 120 seconds prior to measurement. The subsequent cleaning step involves rinsing the sensors in purified water for 10 to 40 s. One sample is measured from 5 to 10 times. Typically, only the last three to four data sets are used for the data evaluation to compensate for potential fluctuations in the intensity of the sensor signals at the beginning of each measurement [56] (Figure 1). It is also reported that samples have been measured seven times, with only the third to fifth measurement [57,58] being selected for further analysis.

The long measurement times of one cycle, including the cleaning steps along with the high number of measurement repetitions for one sample, and the discarding of at least the first dataset explain why the commercial systems are unsuitable for evaluating taste-masking in-line. Further, according to manufacturer specifications, commercial e-tongues require a minimum sample volume of 35 mL (Insent taste sensing systems, Insent Inc., Atsugi-Shi, Japan [59]) and 20 mL (ASTREE liquid and taste analyzer, AlphaMOS, Toulouse, France [60]), respectively. Thus, for each time point of a dissolution experiment at which information about the taste shall be provided, an individual dissolution experiment has to be carried out [39].

As in-line measurements are the most comparable to human taste assessment, such experiments have been performed using lab-made nonspecific, low-selective potentiometric e-tongue-based sensor arrays. These arrays remained in the sample solution throughout the entire time of the drug dissolution process [61,62]. Although the results were promising, the underlying measurement procedure risks bringing sensors into contact with solid particles [40]. As particles could damage polymer-based sensor membranes, suppliers of the commercial systems by Insent Inc. and AlphaMOS recommend analyzing only particle-free/filtered solutions.

## 4. Results and Discussion with Regard to the Performance Qualification

**Figure 2:**
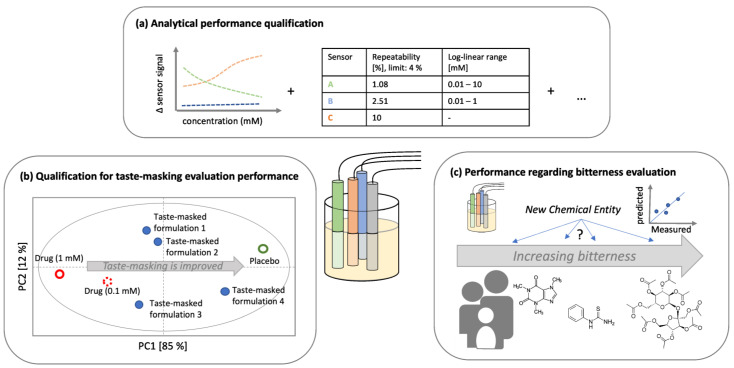
Expectations an e-tongue shall fulfill comprise (**a**) proper analytical performance, (**b**) reliable performance in taste-masking assessment, and (**c**) evaluation of absolute bitterness of new substances.

According to GMP, the PQ allows for the defining of performance criteria and tolerance limits to prove whether the instrument ‘performs as expected under real conditions’. Considering the expectations an e-tongue shall fulfill, this comprises (a) a proper analytical performance, (b) a reliable performance regarding bitterness evaluation, and (c) the evaluation of taste-masking performance (Figure 2). In the following sections, the authors will present and critically discuss what has been presented with regard to (a)–(c) in the reviewed publications of the past decade.

### 4.1. Analytical Performance Qualification

**Figure 3:**
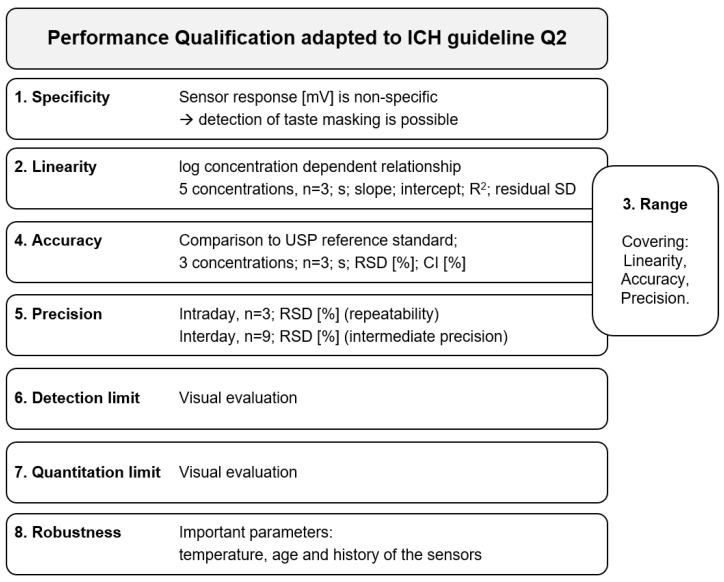
Guidance on qualification of an e-tongue, adapted from [35].

To address a PQ of e-tongues from an analytical point of view, the literature provides two approaches [30,39] that follow the International Conference on Harmonization (ICH) guideline Q2(R1) by [1]. The authors of these publications adapted details where necessary to propose guidance for qualifying the commercial e-tongues by Insent Inc. (Atsugi-Shi, Japan) and AlphaMOS (Toulouse, France) (Figure 3).

For the presented PQ experiments, the authors mainly used quinine hydrochloride to qualify the Insent taste sensing systems (Insent Inc., Atsugi-Shi, Japan) and caffeine citrate to qualify the ASTREE liquid and taste analyzer (AlphaMOS, Toulouse, France). Additionally, to evaluate the non-specificity, other substances were analyzed. In both publications, the authors indicated what has been stated earlier in another context [63], namely, that an ionic character seems to be a prerequisite for a trustworthy taste-masking assessment by e-tongue measurements. With a relative standard deviation (RSD) limit of 4% for intra-day precision, also referred to as repeatability, important guidance for regulatory purposes was suggested [30]. However, the inter-day (intermediate) precision over 6 months did not show acceptable RSD values for any sensor. To solve this issue, it was recommended to make use of an external standard [40], which was also proposed earlier [25]. The obtained Δ signal, e.g., obtained by subtracting the sensor response of quinine hydrochloride in a defined concentration from those of the samples [64], should ensure that the occurring mid-term sensor value variations, also referred to as drift [65], can be adjusted.

This approach was applied in two subsequent studies [39,53]. The first study enhanced the analytical PQ of e-tongues by providing detailed information about the PQ process for the ASTREE liquid and taste analyzer (AlphaMOS, Toulouse, France) [39], while the second study finalized the PQ of the Insent tate sensing systems (Insent Inc., Atsugi-Shi, Japan) by presenting the results of interlab-precision experiments [53]. However, in both studies, the measurement history of the sensors had an impact on sensor responses, irrespective of using a Δ signal. In reference to the latter, the authors hypothesized that, regardless of the applied washing procedure, irreversible binding of certain substances to the lipid membrane could not be prevented. This assumption was recently also discussed in a review [66], where it was generally acknowledged that ‘issues related to spontaneous changes of membrane composition that can occur during [Ion-Selective Membranes] sensor […] operation, seem to be underestimated in the subject literature’. However, from the pharmaceutical regulatory perspective, comprehensive knowledge about sample-dependent sensor behavior should be mandatory for a reliable e-tongue application in formulation development.

In this regard, it should be mentioned that surfactants as well as lipophilic substances contribute to the decreased analytical performance of ion-selective membrane sensors [66]. This finding was attributed to the partitioning of these molecules to the membrane phase and their subsequent accumulation over time. Given that surfactants and lipophilic active pharmaceutical ingredients are prevalent in pharmaceutical formulations, it is highly probable that frequently used e-tongue sensors are commonly exposed to one or both substances. This topic was addressed in a study, where the impact of different samples on the performance of nonspecific, low-selective potentiometric e-tongue sensors was analyzed immediately after each new sample was tested [64]. The samples consisted of various excipients, including a bitter model drug, sodium lauryl sulfate, and a paraben, individually and in combination, to mimic the composition of an oral drug formulation. Following the measurement of each excipient, a concentration series of quinine hydrochloride was analyzed, and the slopes were calculated. These slopes were then compared to slopes obtained from sensors of the same batch that measured a reference solution containing tartaric acid and potassium chloride in between the concentration series (control sensor set). The researchers observed that the impaired sensor performance was evident from significantly altered slopes in the concentration series of the external standard substance. Interestingly, the slopes of regularly used sensors decreased [39,53], while an increase in slope occurred after contact with sodium lauryl sulfate as the sample [64]. As mentioned in ‘A Guide to Ion Selective Measurement’ [67], ‘slope values are often stated in % efficiency terms’. For a pH electrode measuring a monovalent ion, an ideal slope corresponding to 100% slope efficiency would be 59.16 mV at 25 °C, and ‘Slope values below 90% efficiency could indicate electrode contamination’. Applying comparable information to e-tongue sensors would be beneficial. Nonetheless, information relating to still acceptable changes in the sensitivity (slope) of such sensors is lacking. Thus, although users in the pharmaceutical industry might observe changing slopes for predefined drug substances during mid- to long-term usage, action possibilities are lacking as control limits for acceptable slopes are missing. Since the success of a taste-masking strategy is also visible with flat sensor slopes, ‘aged’ sensors (with altered membrane compositions due to the measurement history) can still be used in principle. However, this could lead to different results for the same taste-masking strategy when evaluated at two consecutive time points. This, in turn, prevents the use of e-tongues as a standardized measuring instrument in drug development.

In summary, there are reliable protocols available to qualify the performance of e-tongue instruments from an analytical standpoint. Nevertheless, the ability of the sensors to accurately assess concentration-dependent signals must be closely monitored over the time of usage. Ideally, measures for slope control limits should be identified. This becomes of particular interest with regard to the findings of [68], who discussed the change in concentration-dependent potential, defined as the dose-response slope of the sensor outputs, as a useful bitterness evaluation index.

### 4.2. Qualification for Taste-Masking Evaluation Performance

Taste-masking of a drug formulation is required if two factors of a (per)oral dosage form coincide: the drug substance has an unpleasant taste, and the drug remains in the mouth for a prolonged period. A successful taste-masking strategy is thus needed for solid peroral dosage forms, which are preferred by patients over liquid forms [3], and particularly for orodispersible dosage forms. This is such in the case of orodispersible tablets characterized by a maximum disintegration time of 3 min [69]. A successful taste-masking strategy is thus crucial at least to overcome the 3 min disintegration period before the drug is swallowed, along with the excipients.

Taste-masking evaluation studies are generally based on the assumption that a bitter drug becomes less detectable by a proper taste-masking strategy. This means that, for a taste-masked formulation, the sensor signals of the sensors dedicated to the bitter taste align with the signal values achieved for the dissolved taste-masking substance without the drug. To prove this assumption by e-tongue, ensuring the concentration-dependent behavior of one or more e-tongue sensors in advance within each study is mandatory [30,42].

During the last decade, several studies have focused on the evaluation of taste-masking success in different dosage forms. Some authors assessed the taste-masking performance of various excipients using solutions of the drug and additives, while others examined the final solid drug form. In the following, the experimental setup and findings of the reviewed articles (see Section 2 and Table S1) will be presented and discussed.

#### 4.2.1. Solutions

As, finally, all formulations are analyzed as solutions, this section describes those experiments where the drug substances and possible taste-masking excipients (and thus, no drug formulations) are directly dissolved (Table S1). This procedure allows for the initial assessment of possible interactions of the drug substance with dedicated bitterness sensors. By evaluating the sensitivity of the e-tongue sensors for the drug of interest, a decision can be made about whether an e-tongue can be employed to evaluate a taste-masking success, e.g., by adding different excipients to the solution [5]. In this way, for example, a successful encapsulation of bitter drug molecules in cyclodextrins [70,71,72], liposomes [73], and taste-masking by sweeteners [58] could be demonstrated. In these studies, the drug substances have initially been dissolved in water, citric buffer (pH 4.4), or a water–ethanol mixture. The use of an alcohol–water mixture (30% ethanol) as a dissolution enhancer was specifically employed when drugs with limited water solubility were analyzed [58]. Since the recommended cleaning solutions for the Insent taste sensing systems also contain 30% ethanol, it is not expected to adversely affect the performance of the sensors.

Sweeteners commonly used for taste-masking purposes include sugars as well as lactose, mannitol, maltodextrin, acesulfame, sodium saccharine, or aspartame [5,58,74,75]. To determine the impact of sweet taste-masking additives on the detection of the dispersed drug, the pure drug solutions were analyzed using the e-tongues, and the sensor responses were taken as reference values. Subsequently, the drug solutions were mixed with the taste-masking agents, and the samples were measured [5,58,74]. In studies involving the use of cyclodextrins (specifically hydroxypropyl-β-cyclodextrin) for taste masking [70,71], the cyclodextrin solutions were mixed with the drug solutions in varying ratios to facilitate interaction between the complexes and the drug molecules, or the cyclodextrins were milled together with the drug and the physical mixture was dissolved in water afterward [72]. In one of the mentioned studies, the dispersions were filtered prior to the e-tongue measurements to prevent sensor damage caused by particles [71].

**Figure 4:**
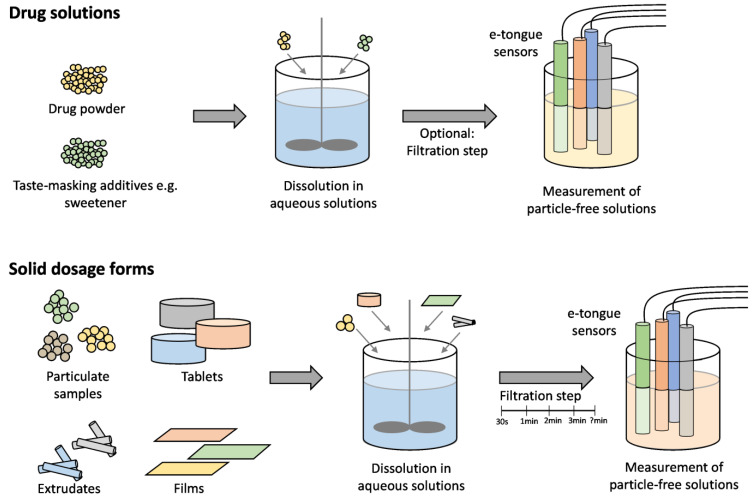
Sample preparation for e-tongue measurements of drug solutions and peroral solid dosage forms.

Highlighting the evaluation of solid oral dosage forms with e-tongues, it has to be considered that particles can potentially damage the sensors [10]. This necessitates particle removal through a filtration step prior to the measurement procedure (Figure 4).

As most utilized e-tongue systems are only sensitive to ionized substances, interpreting the taste-masking effect of non-ionized substances, such as lactose and mannitol, typically requires critical expert knowledge. In this regard, it has already been discussed whether the sweetness of viscosity-enhancing but non-charged substances could be indirectly applied to evaluate sweetness [76]. Further, sensors applied for detecting the sweetness of non-charged sugars, as well as negatively charged high-potency sweeteners and positively charged high-potency sweeteners, have been presented (summarized inter alia by [77]). These developments take into account the chemical diversity of sweeteners. However, even charged artificial sweeteners are not solely dedicated to sweetness, as they are also known for their partially unpleasant taste [78].

In addition to this, alcohols, which are added to improve the dissolution of hydrophobic drug substances, should be used with care. Considering the physiological conditions in the human oral cavity, the drug (formulation) is exposed to saliva. Human saliva has a pH ranging from 6.24 to 7.36 and consists mainly of water, with low concentrations of proteins like mucin (approximately 1.0 to 1.4 mg/mL) and various dissolved electrolytes [79]. If a drug is administered to a patient and taste-masking is required due to an unpleasant taste, enhancing the drug’s solubility will not accurately reflect the actual conditions in the oral cavity. Increased solubility of the drug would result in comparably stronger taste experience, as if the drugs is only partially dissolved. This, in turn, may underestimate the taste-masking success of added excipients in the analytical study. Therefore, the use of solubility enhancers should be approached with caution and may only provide limited information for taste-masking success.

#### 4.2.2. Powders, Microspheres, and Granules

The powders or microspheres considered for this review (Table S1) were mainly prepared through spray drying. In these cases, the bitter-tasting drug was formulated with taste-masking polymers, such as various types of Eudragit^®^ [57,80], two types of Kollicoat^®^ [81] or carrageenan [82], cellulose derivates [83], or cation-exchange resins [84]. In one study, taste masking was achieved by using nanoemulsions of different oils. The drug Ibuprofen was dissolved in each oil separately, emulsified in water, and subsequently spray-dried, with maltodextrin serving as the matrix material and taste-masking additive [56].

Furthermore, taste-masking experiments have also been carried out by preparing granules. In these cases, drug substances were coated onto pellets in a fluidized bed [85,86] or mesoporous silica carrier-based composites were used to embed the drug within the porous structure [87]. Prior to taste-masking assessments, these pellets were further embedded in release-regulating matrices, e.g., by hot melt extrusion [87], coating the pellets with water-soluble films [86] or controlled-release films [85].

Samples for e-tongue measurements were typically prepared as follows: the powders were dissolved in from 5 to 100 mL of water or artificial saliva buffer solution for a duration of from 30 to 300 s [56,57,84,87]. A filtration step was then performed using 0.45 µm membrane filters to remove insoluble components or any remaining particles, ensuring a particle-free solution [57,84] (Figure 4). When dried emulsions were employed as a taste-masking strategy, it was assumed that the drug molecules would predominantly remain in the oil phase, preventing direct contact with the oral cavity. Prior to evaluating the taste-masking effect, the dried emulsion powders were redispersed in water, leading to the release of droplets with a size in the low micrometer range [56]. Since no further information was provided on how the samples were prepared, it was assumed that the emulsions were directly measured with the e-tongue. For evaluating the taste-masking effect in prolonged-release dosage forms, samples were added to distilled water and stirred for up to 60 min before being filtered and measured [85]. However, since these solid drug formulations are not intended to be kept in the mouth for so long, the related results are only partially relevant for taste-masking evaluation purposes.

To better reflect the actual intake behavior of patients regarding powders, microspheres, or granules, it can be assumed that these dosage forms are predominantly swallowed directly with some liquid, or possibly sprinkled over food. In the case of further processing into tablets, these would also be swallowed directly and not remain in the oral cavity for an extended period. When evaluating the taste-masking experience under realistic conditions, it is important to consider the dissolution time in the aqueous solution. However, if a comparison between different taste-masking formulations is desired, a sample disintegration and drug dissolution time of approximately 180 s seems to be appropriate.

#### 4.2.3. Orodispersible (Mini-)Tablets

In most cases, powders are not administered directly to the patient but are further processed into tablets. Orodispersible tablets (ODTs) are considered beneficial dosage forms, especially for children. Consequently, many publications assess taste-masking success for these dosage forms using e-tongue analysis (Table S1). ODTs have been formulated by incorporating bitter-tasting drugs in ethylcellulose matrices [88,89] or Eudragit matrices [55,90]. Additionally, they have been developed by mixing the drugs with sweeteners, flavors, or other additives [89,91,92]. Alternatively, the bitter taste has been masked by adding either cyclodextrins [93] or utilizing ion exchange resins [93,94]. The ODTs themselves were mainly prepared by tableting, freeze-drying, or 3D-printing techniques.

ODTs should disintegrate in water within 3 min [69]. In the reviewed studies, from 1 to 20 units of ODTs were dispersed in from 10 to 100 mL of distilled water for 30 s or 3 min [55,89,91,92] (Figure 4). Prior to the e-tongue measurement, the samples were filtered; however, the duration of the filtration step has only been mentioned infrequently. For a credible assessment of the taste-masking success of the above formulations, however, it would be extremely useful to standardize the filtration step prior to the e-tongue measurements. The active drug substance is still being released during the filtration. This, in turn, could have a direct influence on the e-tongue results. Therefore, the duration of filtration should be considered for the e-tongue performance qualification [95].

#### 4.2.4. Other Solid Dosage Forms

In addition to the aforementioned dosage forms, other solid formulations have also been investigated, including extrudates from hot-melt extrusion [96,97,98], (mini-)tablets [99,100], orodispersible and mucoadhesive films [101,102], nanofibers from co-axial electrospinning [103,104], and SNEDDS-formulations [105].

For hot-melt extrudates, taste-masking strategies primarily involved the use of different types of Eudragit polymers. Nanofibers were taste-masked with both Eudragit polymers and cyclodextrins. Cyclodextrins were also further investigated for the formulation of orodispersible and mucoadhesive films, with [102] the additional utilization of maltodextrin in their films. The mini-tablets, intended for administration to cats, employed L-lysine as a flavoring agent [99].

The formulations discussed in this section are very heterogeneous. Still, all of them were described to be initially dissolved in aqueous solutions from 15 to 60 s. Except for the mini-tablets, which were ground in a mortar first [99], each dosage form was put into the dissolution liquid unmodified. The applied dissolution media were aqueous potassium chloride solutions [97,99,103,105], buffer solutions at pH 6.8 [96], or deionized water [98,102] in volumes of 25–100 mL. Whether the samples were filtered prior to the following e-tongue measurements was not always mentioned.

#### 4.2.5. Indicators for Successful Taste-Masking Performance

Reviewing the studies published during the last decade (Table S1), it was found that 26/55 projects presented in vivo data, human or rodent, to validate e-tongue results. Throughout the projects, 36 drug substances were evaluated. Cetirizine hydrochloride salts were stated in four studies, berberine hydrochloride salts in three studies, and rupatadine fumarate in two studies. Together with ebastine and chlorpheniramine maleate, eight projects dealing with H1-Antihistamins were identified. It was concluded that correlations between in vivo and e-tongue data on taste-masking success worked well, particularly for ionized drugs and very particularly for drugs with chemical properties comparable to H1-Antihistamins. Difficulties arose for non-charged substances [106].

Two projects presented a successful taste-masking strategy for propranolol hydrochloride (HCl) [72,98]. A comparable approach seemed to be the basis for supporting the taste-masking strategy in an EMA report on the human use of the preparation Hemangiol^®^. The authors stated that the e-tongue results for the formulation containing propranolol HCl ‘demonstrated ~80% masking effect compared to the reference in water’ and that in the following ‘neither signals of poor acceptability nor premature treatment discontinuation were observed for reasons of taste during clinical studies’ [107].

It can thus be summarized that e-tongues are qualified to perform taste-masking evaluation properly, at least for some projects and only for distinct drugs.

### 4.3. Performance Regarding Bitterness Evaluation

From an evolutionary point of view, it is assumed that bitterness prevents mammals from intoxication (Lindemann, 1996). The taste impression is initiated by a substance that couples to at least one of the ~25 *h*TAS2 G protein-coupled receptors, which are the bitter taste receptors located on the human tongue [108,109]. These substances include toxic plant metabolites as well as synthetic compounds and are chemically diverse, comprising acetylated sugars, alkaloids, amides, amines, amino acids, azacycloalkanes, carbamides, carbonyl compounds, esters, fatty acids, flavonoids, glycosides, lactones, metal ions, N-heterocyclic compounds, peptides, phenols, secoiridoids, steroids, terpenoids, thioureas, and ureas. Taste cells, however, also identify toxic chemicals only as partially bitter, as indicated by bitter chemicals like caffeine or quinine [110]. The same holds true for a large portion of drugs. To evaluate whether drugs, especially NCEs, would result in an activation of the TAS2R, an analytical solution is needed. However, contrary to sweet substances with the common structural features of a proton-donor/proton-acceptor system combined with a hydrophobic group, bitter compounds cannot be summarized by such universal structure elements [111]. Although the hydrophobicity of chemical compounds correlates with the intensity of the bitter taste [112], and their surface tension seems to have a reciprocal relation following Szyszkowski’s equation [113], bitterness is, in sum, a complex reaction which several receptors could contribute to and which is not yet fully decoded [108].

Given the complexity of bitterness, it is a challenge for an e-tongue to provide reliable information about bitterness. This became apparent in a study from 2006 [41]. Researchers performed a study with the ASTREE liquid and taste analyzer (AlphaMOS, Toulouse, France), whereby one objective was ‘to investigate the potential use of e-tongue in ranking relative bitterness of compounds’. To do so, they aimed to assess the possible bitterness of the drug substances by applying principal component analysis (PCA) and the resulting group distances (Euclidean distances). This methodology became common in this field (see beside others [42,114,115]. The assumption was that higher group distance values between pure water and the drug-containing solutions indicate a greater level of bitterness for the drug substances. Each drug substance was analyzed by [41] as an aqueous solution of 10 mM, and the results suggested the following bitterness ranking (less bitter from left to right):

ranitidine HCl > prednisolone Na > quinine HCl ∼ phenylthiourea > paracetamol >> sucrose octaacetate > caffeine

Curious about the ‘web-server for the prediction of organoleptic properties of chemical compounds’ [116], which is one of the most used and tested databases and particularly contains a great variability of bitter compounds [117], the authors of the current review used this webserver to compare the aforementioned drugs in terms of bitterness. It was found that this program calculated the probability of the assessed substance being bitter. A similar probability evaluation for the taste impressions would also be possible for sour and sweet. The probability ranking for the bitterness of the aforementioned drugs is the following, with 1.000 indicating the highest probability:

quinine HCl (1.000) > caffeine (0.999) > phenylthiourea (0.966) > prednisolone (0.908) > paracetamol (0.861) > ranitidine HCl (0.737)

Unfortunately, it was not possible to obtain information for prednisolone as a sodium salt, only for prednisolone as a base. Since this web-based probability calculation only provides information on whether a substance is expected to be bitter, the lack of correlation between these data and the e-tongue data, which aims to rank bitterness, may not be too questionable at first glance. However, it should be mentioned that first, ranitidine HCl, which was detected by the e-tongue as the most bitter substance, is only predicted with a probability of 0.737 to be bitter. Second, sucrose octaacetate is missing in the probability ranking because it was predicted to be sweet with a probability of 0.752. Though the bitter prediction failed, this is refuted, as sucrose octaacetate is well-known to be a ‘bitter acetylated sugar’ [118].

Emphasizing the fact that information about human taste sensation is essential to assessing instrumental taste sensing or data-based calculations, published human taste threshold data were gathered. Unfortunately, the authors were unable to find human threshold data for ranitidine HCl and prednisolone sodium. Indeed, publications can be found dealing with the taste intensities of, e.g., prednisolone [119] or ranitidine hydrochloride [120] compared to other drugs. However, for comparing the results of different experimental studies, the molecules should be the same (only comparing the same salts), and information about identified thresholds is required. This information could not be identified in the literature. This is why the authors excluded information for ranitidine HCl and prednisolone sodium from the following discussion. However, taste threshold data for caffeine, paracetamol, phenylthiourea, quinine HCl, sucrose octaacetate, and several other drugs (Table 2) were collected.

To arrange the human thresholds based on the mean literature values, the thresholds were ordered as follows (from left to right the higher the threshold concentration):

sucrose octaacetate ≤ quinine HCl < phenylthiourea <<< caffeine < paracetamol

**Figure 5:**
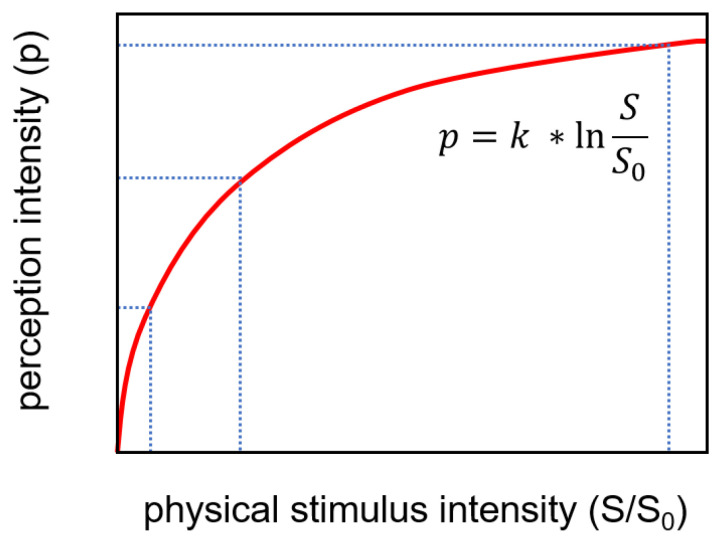
Graphical illustration of the Weber–Fechner-law.

This sequence is also not comparable to the ranking of predicted bitterness by the e-tongue as proposed by [41]. However, it must be mentioned that samples of the e-tongue assessment were conducted using significantly higher concentrations (10 mM) compared to the concentrations used for determining human thresholds. This could have the consequence that even differences in human perception might be low, as the intensity of the perception tends to decrease with increasing concentration (Figure 5).

Furthermore, the comparability of the e-tongue ranking with other rankings is also debatable, from our point of view, particularly in the discussed study where non-ionic drugs (phenylthiourea, paracetamol, and caffeine) were ranked against ionic drugs, which can be detected much better by e-tongue systems.

The authors are aware that there are numerous approaches for bitterness prediction/evaluation based on physicochemical characteristics that have been published in the literature, e.g., [68,127,128]. However, approaches that correlate human data with e-tongue data were found to be the most meaningful. Therefore, some further projects dealing with this approach will be discussed.

In 2011, researchers from the School of Pharmaceutical Sciences of Mukogawa Women’s University in Japan, in collaboration with scientists of Insent Inc., introduced a new bitterness sensor for the taste sensing systems (Insent Inc., Atsugi-Shi, Japan), called SB2BT0 [129]. They analyzed solutions of quinine hydrochloride as well as eight charged H1-receptor antagonists and compared the data obtained from the SB2BT0 sensor to those obtained from a human taste panel [130]. Therefore, 11 volunteers were initially trained by assigning quinine HCl solutions of 0.01, 0.03, 0.10, 0.30, and 1.00 mM to related bitterness scores. They were then asked to assess the bitterness of provided test sample solutions based on the trained bitterness scores. To predict the bitterness intensities of the H1-receptor antagonists, a multiple regression analysis was performed using the SB2BT0 sensor signal, its CPA value, and the adsorption ability (CPA/sensor signal). After optimization, these data correlated well with the results obtained from the human taste panel (R^2^ = 0.910).

A similar correlation was published utilizing the ASTREE liquid and taste analyzer (AlphaMOS, Toulouse, France). There, the same eight H1-receptor antagonists were evaluated using seven sensors (labeled ZZ, AB, GA, BB, CA, DA, and JE), along with a human sensory panel consisting of 11 trained volunteers [131]. In this study, the volunteers were also asked to compare the bitterness of the H1-receptor antagonists (each at a concentration of 0.1 mM) to the bitterness perception of one of the tested quinine hydrochloride solutions with concentrations of 0.01, 0.03, 0.1, 0.3, and 1 mM. The same samples evaluated by the human panelists were analyzed using the e-tongue. Researchers identified sensors AB and JE as being best suited for bitterness evaluation. Based on the responses of these sensors and the results of the bitterness scores evaluated by the human panel, a partial least squares regression analysis was conducted. The predicted bitterness values from the e-tongue showed high agreement with the actual human scores (R^2^ = 0.9621).

Both studies provide encouraging evaluations, but, due to the similar physicochemical properties of the investigated drugs (Table 3), this is rather an evaluation than a prediction approach.

However, such an evaluation approach could also support a formulation strategy for a new chemical entity, as presented in 2017 [5]. Instead of relying solely on trained or untrained volunteers for taste evaluation, researchers implemented taste-related questions in a phase I study (single-center, double-blinded, randomized, placebo-controlled, single ascending dose) in healthy subjects [5]. Simultaneously, the NCE was analyzed at different concentrations using a successfully qualified [30] Insent SA402B e-tongue. A comparison between the human and e-tongue data indicated that the sensory attributes of ‘unpleasant taste’, ‘bitterness’, and ‘taste intensity’ could be correlated with sensor signals from SB2BT0 and SB2AC0, which are dedicated to cationic bitterness. These findings aligned well with the given information about the chemical properties of the substance (pKa = 10.9) and the applied dissolution medium (citric buffer pH 4.4). As a result, the e-tongue data were qualified in terms of reliable taste data and subsequently utilized to identify suitable taste-masking agents.

Thus, for this last performance aspect, it can be concluded that the evaluation of bitter compounds by e-tongues, rather than a prediction, can be successful; however, correlation with human data is still required for trustworthy results so far.

## 5. Conclusions

Following the definition in the technical report of IUPAC, which describes e-tongues as instruments comprising arrays of nonspecific, low-selective (chemical) sensors with overlapping signals, various literature-known qualified designs have been presented. Additionally, different system checks have been presented as evidence to demonstrate the qualification of e-tongues’ operation principles. When assessing the performance of e-tongue systems, three main tasks are highlighted: their analytical performance, their ability to evaluate taste-masking success, and their capacity to assess or predict actual bitter values.

In summary, e-tongue instruments perform well as analytical instruments and reliable protocols are available to assess their analytical performance. However, systematic research is needed to understand the impact of sensor history and age, which can lead to changes in sensor sensitivities. The reviewed projects utilizing e-tongues for taste-masking assessment have demonstrated the general applicability of these instruments. Yet the most reliable results are typically obtained for ionized substances. Furthermore, when aiming to achieve results that align with human taste sensation, careful consideration must be given to the sample preparation process, including the choice of solvents as well as the duration of dissolution and filtration for solid oral dosage forms.

Regarding bitterness evaluation by e-tongues, rather than prediction, it has shown promising success. However, to obtain trustworthy results, further correlation with human data is necessary since the biological taste system is too complex to be fully modeled and predicted solely by an artificial sensor.

## Figures and Tables

**Table 2:** Published taste threshold data for different drug substances.

Drugs	Human Thresholds [mM] *	Reference
caffeine	**1.2** *(detection threshold, ø age of panellists 23 y)*	[121]
	**2** *(detection threshold, ø age of panellists 26 y)* **2.9** *(recognition threshold, ø age of panellists 26 y)*	[122]
	**0.7**	[123]
diphenhydramine	**1**	[114]
efavirenz	**0.039**	[105]
paracetamol	**2**	[124]
phenylthiourea	**0.049** *(taste threshold of panellists aged 10–29 y)*	[125]
	**0.02***(tasters)* **8** *(non-tasters)*	[123]
quinine HCl	**0.0083** *(detection threshold, ø age of panellists 23 y)*	[121]
	**0.0048** *(detection threshold, ø age of panellists 26 y)* **0.0087** *(recognition threshold, ø age of panellists 26 y)*	[122]
	**0.03**	[123]
quinine sulphate	**0.008**	[123]
	**0.012**	[126]
sucrose octaacetate	**0.0036/0.0098** (*detection threshold, ø age of panellists 26/88 y)* **0.0068/0.05** *(recognition threshold, ø age of panellists 26/88 y)*	[122]
	**0.004**	[118]

* for a better comparability, found thresholds were all converted into mM concentrations.

**Table 3:** Physicochemical properties of quinine hydrochloride dihydrate and eight H1-receptor antagonists; bitterness score by sensory testing data and Euclidean Distance to water as well as the pH (0.1 M) values taken from [121], pKa-, logP-, and M_R_-values taken from drugbank.com, and the respective accession numbers; HCl = hydrochloride.

Drug	Bitterness Score by Sensory Testing	Euclidean Distance to Water	pH (0.1 M)	pKa (Strongest Basic)	logP *	M_R_ [g/mol]	Drugbank Accession Number (https://go.drugbank.com/ assessed on 8 May 2024)
Quinine HCl dihydrate	2.00	376.70	5.43	9.05	2.82 2.51	396.9	DBSALT001044
Cetirizine HCl	0.36	1258.78	3.76	7.74	2.98 0.86	461.8	DBSALT001214
Diphenhydramine HCl	0.45	495.33	5.20	8.87	3.44 3.65	291.8	DBSALT000056
Chlorpheniramine maleate	1.00	412.92	5.30	9.47	3.74 3.58	390.9	DBSALT000987
Epinastine HCl	1.82	300.63	5.32	9.31	2.34 3.13	285.8	DBSALT000961
Ketotifen fumarate	4.38	681.45	4.38	7.15	3.49 3.35	425.5	DBSALT001856
Olopatadine HCl	4.23	943.42	4.23	9.76	3.99 0.75	373.9	DBSALT000685
Fexofenadine HCl	1.18	945.68	4.30	9.01	5.02 2.94	538.1	DBSALT001227
Azelastine HCl	5.10	3.27	5.10	8.88	3.81 4.04	418.4	DBSALT000013

* first value as provided by ALOGPS, second value as provided by ChemAxon. Both values were taken as listed by https://go.drugbank.com/ (assessed on 8 May 2024).

## Data Availability

Not applicable.

## Supplementary Materials

The following supporting information can be downloaded at the following location: https://www.mdpi.com/article/10.3390/pharmaceutics16050658/s1, Table S1: Summarized information about References found in between 2014–2023. References [132,133,134,135,136,137,138,139,140,141,142,143,144,145,146,147,148,149,150] are cited in the supplementary materials.
