# Supplementary material for: Critical View on the Qualification of Electronic Tongues Regarding Their Performance in the Development of Peroral Drug Formulations with Bitter Ingredients
Source: Pharmaceutics. 2024 May 15;16(5):658. doi: 10.3390/pharmaceutics16050658 (PMC11125162; doi:10.3390/pharmaceutics16050658)
Supplement: Supplementary file 1 [file pharmaceutics-16-00658-s001.zip › pharmaceutics-2959206-supplementary.pdf]

Supporting information

Critical view on the qualification of electronic tongues regard-ing their performance in the development of peroral drug for-mulations with bitter ingredients

Denise Steiner[1], Alexander Meyer[2], L. Isabell Immohr[3], Miriam Pein-Hackelbusch[2,]*

[1]Institute of Pharmaceutical Technology and Biopharmaceutics, University of Muenster, Corrensstraße 48, 48149 Muenster, Germany
[2]Institute for Life Science Technologies (ILT.NRW), Ostwestfalen-Lippe University of Applied Sciences and Arts, Campusallee 12, 32657 Lemgo, Germany
[3]NextPharma Germany Bidco GmbH, Hildebrandstrasse 12, 37081 Goettingen, Germany

Table S1: Summarized information about References found in between 2014-2023; CPA = change in membrane potential due to adsorption, PCA = principal component analysis, PLS = partial least squares regression, DFA = discriminant function analysis, BATA = brief aversion taste assessment – performed with rodents, NCE = new chemical entity; HME = hot melt extrudates; ODT = orodispersible tablets; ODF = orodispersible films

Year	Applied electronic tongue	Taste-masking evaluation by					Analysed drugs	Formulation	Refe-rences
		Dedicated sensors	CPA value	(Statistical) analysis of electronic tongue data	Human data	Others			
2014	αASTREE II			PCA			Acetaminophen<br>Ibuprofen<br>Tramadol HCl<br>Sildenafil citrate	Solution	[58]
	αASTREE II			PCA	x		Ebastine	ODT	[132]
	Insent TS-5000Z<br>αASTREE II	SB2BT0		PCA and PLS	x		Propranolol HCl	HME	[98]
	Insent TS-5000Z<br>αASTREE II			PCA			Loperamide HCl<br>Dextromethorphan HBr	Solution	[133]
	αASTREE II			PCA and DFA	x		Sildenafil citrate	Microcapsules	[57]
2015	Insent TS-5000Z			PCA			Diclofenac<br>Diclofenac sodium<br>Diclofenac potassium	ODT	[55]
	αASTREE			PCA and PLS	x		Famotidine<br>Amlodipine besylate	ODT	[89]
	Insent TS-5000Z			PCA	x		Cetirizine HCl	ODT	[93]
2016	ASTREE			PCA			Ketoprofen	HME	[96]
2017	Laboratory prototype potentiometric e-tongue			PCA	x		Cetirizine 2HCl	Powder	[80]
	Laboratory prototype potentiometric e-tongue			PCA	x		Cetirizine 2HCl	ODT,<br>Lyophilisates	[90]
	αASTREE			PCA, model based on e-Tongue Euclidean distance measurements, Ka and Kd of the drug/cyclodextrin-complexes			44 bitter drugs	Solution	[71]
	Insent TS-5000Z			PCA	x		Two NCEs	Solution	[5]
	ASTREE			Bitterness score *(calculated with electronic tongue software)*			Mefloquine HCl	Liposomes	[73]
	Laboratory prototype potentiometric e-tongue			PCA	x		Cetirizine 2HCl	Microspheres	[81]

Year	Applied electronic tongue	Taste-masking evaluation by					Analysed drugs	Formulation	Refe-rences
		Dedicated sensors	CPA value	(Statistical) analysis of electronic tongue data	Human data	Others			
2018	Insent TS-5000Z			PCA			Ranitidine HCl	Solution	[134]
	ASTREE II			PCA	x		Traditional chinese herbs	Solution	[135]
	ASTREE II			PCA	x	BATA	Berberine HCl	Minitablets	[100]
	Insent TS-5000Z	SB2AC0<br>SB2AN0<br>SB2C00<br>SB2AE1		PCA		BATA	Isoniazide	HME	[97]
	Laboratory prototype electrochemical impedance spectroscopy-based e-tongue			multidimensional projection techniques such as Sammon's Mapping (SAMMON) and PCA but with Interactive Document Map showing the best results			Praziquantel	Microcapsule s	[136]
	Insent SA402B	SB2BT0		PCA			Quinine HCl x 2 H_2O	HME	[137]
2019	Insent TS-5000Z	SB2C00<br>SB2AE1<br>SB2AC0<br>SB2AN0		Sensor signal			Chlorpheniramine maleate	Solution	[138]
	ASTREE II			PCA			Rhei radix et rhizome<br>Berberine HCl<br>Scutellaria baicalensis	Powder	[139]
	αASTREE			PCA	x		Nizatidine	Powder	[84]
	Laboratory prototype voltammogramm-based e-tongue			PCA	x		Efavirenz	SNEDDS	[105]
	Laboratory prototype potentiometric e-tongue			PCA	x		Rupatadine fumarate	Powder	[83]
2020	Insent TS-5000Z	SB2BT0<br>SB2AN0<br>SB2C00<br>SB2AE1	x	Sensor signal		for veteri-nary appli-cations	Amlodipine besylate	Minitablets	[99]
	Insent TS-5000Z	SB2BT0<br>SB2AN0<br>SB2C00<br>SB2AE1	x	PCA			Cefpodoxime proxetil	Solution	[140]
	Insent TS-5000Z	SB2AC0<br>SB2AN0<br>SB2C00<br>SB2AE1		Sensor signal	x	BATA	Isoniazid<br>Rifampicin<br>Pyrazinamide<br>Ethambutol 2HCl	Solution	[106]
	Insent SA402B	SB2BT0<br>(SB2AN0<br>SB2C00<br>SB2AE1)	x	PCA	x		Atomoxetine HCl	Solution	[70]
	Insent SA402B	SB2AC0<br>SB2AN0	x	*bitterness intensity* estimated as:<br>bitterness intensity 1 = 1.26 × CPA (AC0),<br>bitterness intensity 2 = 1.80 × CPA (AN0)<br>*bitterness scores* calculated on bitterness intensities<br>*total bitterness = (bitterness score 1 + bitterness score 2)/2*			Propranolol HCl	Microbeads	[141]

	Laboratory prototype potentiometric e-tongue			PCA	x		Rupatadine fumarate	ODT	[88]

Year	Applied electronic tongue	Taste-masking evaluation by					Analysed drugs	Formulation	Refe-rences
		Dedicated sensors	CPA value	(Statistical) analysis of electronic tongue data	Human data	Others			
2021	Insent TS-5000Z	SB2BT0	X	PCA			Chlorpheniramine maleate	Nanofibres	[103]
	Insent TS-5000Z	SB2AE1<br>SB2CT0<br>SB2AC0<br>SB2AN0<br>SB2C00		PCA			Indomethacin<br>Furosemide	ODF	[101]
	Laboratory prototype potentiometric e-tongue			PCA			Ketoprofen lysine	Solution	[142]
	Laboratory prototype electrochemical impedance spectroscopy-based e-tongue			Interactive Document Map (IDMAP)		BATA	Artesunate-Mefloquine<br>Praziquantel, Benznidazole	Tablets	[16]
	αASTREE			Bitterness level (response value R)	x	BATA	Ibuprofen	Emulsion	[56]
	Insent TS-5000Z	SB2C00		Bitterness intensity (single sensor response)			Caffeine	Powder	[143]
	ASTREE			PCA, PLS	x		Levetiracetam	ODT	[91]
	Laboratory prototype taste biosensor (rat cardiomyocytes + microelectrode assay)			Multiple feature extraction and parameter visualization for high-content analysis			Denatonium benzoate<br>Quinine<br>Dephenidol<br>Arbutin	Solution	[144]
2022	ASTREE II			PCA			Levocetirizine 2HCl	Nanofibres	[104]
	Laboratory prototype potentiometric e-tongue			Supervised LDA			Ranitidine HCl	Solution	[74]
	Laboratory prototype potentiometric e-tongue			PCA	x		Rupatadine fumarate	ODFs	[145]
	cTongue; Shanghai Baosheng, Shanghai, China			PCA			Berberine<br>Phillyrin	Solution	[146]
	Insent SA402B	SB2AE1<br>SB2CT0<br>SB2AAE<br>SB2CA0<br>SB2C00<br>SB2GL1		Peptides were dissolved in water and added to an 1 mM quinine HCl solution – bitterness intensity reduction activity was calculated based on the Δ sensor responses against quinine HCl as blank control and a particular peptide (LEGSLE) as positive control			Nebulin-derived peptides	Solution	[147]
	Insent TS-5000Z	SB2BT0<br>SB2AN0<br>SB2C00<br>SB2AE1	x	Sensor signal	x		Oseltamivir phosphate	HME	[87]
2023	Laboratory prototype potentiometric e-tongue			PCA			Rosuvastatin calcium	Granules (pellets)	[86]
	ASTREE			Relative distances	x		Azithromycin	Granules (pellets)	[85]
	Insent TS-5000Z	SB2C00<br>SB2AE1<br>SB2AN0	x	Sensor signal			Carbamazepine	ODT	[92]

Year	Applied electronic tongue	Taste-masking evaluation by					Analysed drugs	Formulation	Refe-rences
		Dedicated sensors	CPA value	(Statistical) analysis of electronic tongue data	Human data	Others			
	Insent SA402B	umami,<br>saltiness, sourness,<br>bitterness	x	Random Forest, backpropagation neural network, K-nearest neighbour, support vector machine		uf-GC e-nose	Bear pile powder	Powder	[148]
	Insent TS-5000Z	SB2C00<br>SB2AN0<br>SB2BT0<br>SB2AE1	x	Bitter value	x		Berberine HCl	Solution	[75]
2023	Insent SA402B	SB2AN0	x	Sensor signal	x		Ibuprofen<br>Flurbiprofen<br>Naproxen	Solution	[149]
	Insent SA402B	SB2CA0<br>SB2C00<br>SB2AE1<br>SB2AAE<br>SB2CT0	x	PCA			Lisdexamfetamine	Tablets	[150]
	Insent SA402B	SB2BT0	X	Sensor signal			Diphenhydramine HCl	Granules	[82]
	Insent SA402B	SB2AC0<br>SB2AN0<br>SB2C00<br>SB2AE1		PCA and calculation of bitterness scores			Propranolol HCl	Solution	[72]

Klicken oder tippen Sie hier, um Text einzugeben.
